# Study About Beauvericin and Enniatins: Method Validation and Survey for Foods in Japan

**DOI:** 10.14252/foodsafetyfscj.D-25-00018

**Published:** 2025-12-19

**Authors:** Tomoya Yoshinari, Hiroshi Takeuchi, Masaru Taniguchi, Toru Fukumitsu, Eiko Sato, Shunsuke Zama, Akira Shimoyama, Mamiko Goto, Takashi Morita, Ohnishi Takahiro

**Affiliations:** 1 Division of Microbiology, National Institute of Health Sciences, Tonomachi 3-25-26, Kawasaki-ku, Kawasaki, Kanagawa 210-9501, Japan; 2 Food Safety Research Center, Gifu Prefectural Research Institute for Health and Environmental Sciences, Nakafudogaoka 1-1, Kakamigahara, Gifu 509-0105, Japan; 3 Food Department, Nagoya City Public Health Research Institute, Sakurazaka 4-207, Moriyama-ku, Nagoya, Aichi 463-8585, Japan; 4 Chemistry Division, Kanagawa Prefectural Institute of Public Health, Shimomachiya 1-3-1, Chigasaki, Kanagawa 253-0087, Japan; 5 Kawasaki City Institute for Public Health, Tonomachi 3-25-13, Kawasaki-ku, Kawasaki, Kanagawa 210-0821, Japan; 6 Japan Food Research Laboratories, Nagayama 6-11-10, Tama, Tokyo 206-0025, Japan; 7 Japan Food Inspection Corporation, Heiwajima 4-1-23, Ota-ku, Tokyo 143-0006, Japan; 8 Food Analysis Technology Center SUNATEC, Akahorishinmachi 9-5, Yokkaichi, Mie 510-0825, Japan; 9 Japan Grain Inspection Association, Shiohama 1-2-1, Koto-ku, Tokyo 135-0043, Japan

**Keywords:** beauvericin, enniatin, emerging mycotoxin, method validation, survey

## Abstract

Beauvericin (BEA) and enniatins (ENNs) are cyclic depsipeptide mycotoxins mainly produced by *Fusarium* species. To investigate their presence in retail foods in Japan, we developed an analytical method for the simultaneous determination of BEA, enniatin A (ENNA), enniatin A_1_ (ENNA_1_), enniatin B (ENNB), and enniatin B_1_ (ENNB_1_). Five mycotoxins were extracted from food samples using a mixture of acetonitrile and water, and then purified using a C18 cartridge. LC-MS/MS was used to quantify the purified mycotoxins. This method was validated in an inter-laboratory study. Eight laboratories participated in the study, and three spiked and two naturally contaminated wheat samples were analyzed. The ranges of the mean recoveries of BEA, ENNA, ENNA_1_, ENNB, and ENNB_1_ were 92‒94, 94‒96, 97‒98, 98‒99, and 98‒100%, respectively. The relative standard deviations for repeatability and reproducibility in spiked and naturally contaminated samples ranged from 2.1 to 5.7% and from 6.2 to 15.3%, respectively. After the application of the method to the analysis of these five mycotoxins in other foods was confirmed by recovery tests, 658 food samples including cereals, bean products and dry fruits were analyzed using the developed analytical method. BEA, ENNA, ENNA_1_, ENNB, and ENNB_1_ were detected in 23%, 7%, 17%, 40% and 33% of all samples, respectively, at >1.5 µg/kg. About the result of BEA, the highest mean level in 12 food groups was shown in soybean flour samples (13 µg/kg). Among the four ENNs, the positive rate of ENNB was the highest in all food groups. ENNB was mainly detected in rye flour and wheat flour, and the mean ENNB levels of rye flour and wheat flour were 987 and 49 µg/kg, respectively. Our results are useful for the risk assessment of BEA and ENNs in retail foods in Japan.

## Introduction

The presence and toxicity of emerging mycotoxins in foods and feeds are considered potential threats to human and animal health^1^^)^. Emerging mycotoxins are generally described as mycotoxins that are neither routinely determined nor legislatively regulated; however, evidence of their incidence is rapidly increasing. For example, beauvericin (BEA), enniatins (ENNs), *Alternaria* toxins, moniliformin, and sterigmatocystin are regarded as emerging mycotoxins, and the European Food Safety Authority (EFSA) performed risk assessments for these mycotoxins^2‒5)^.

**Fig. 1.**
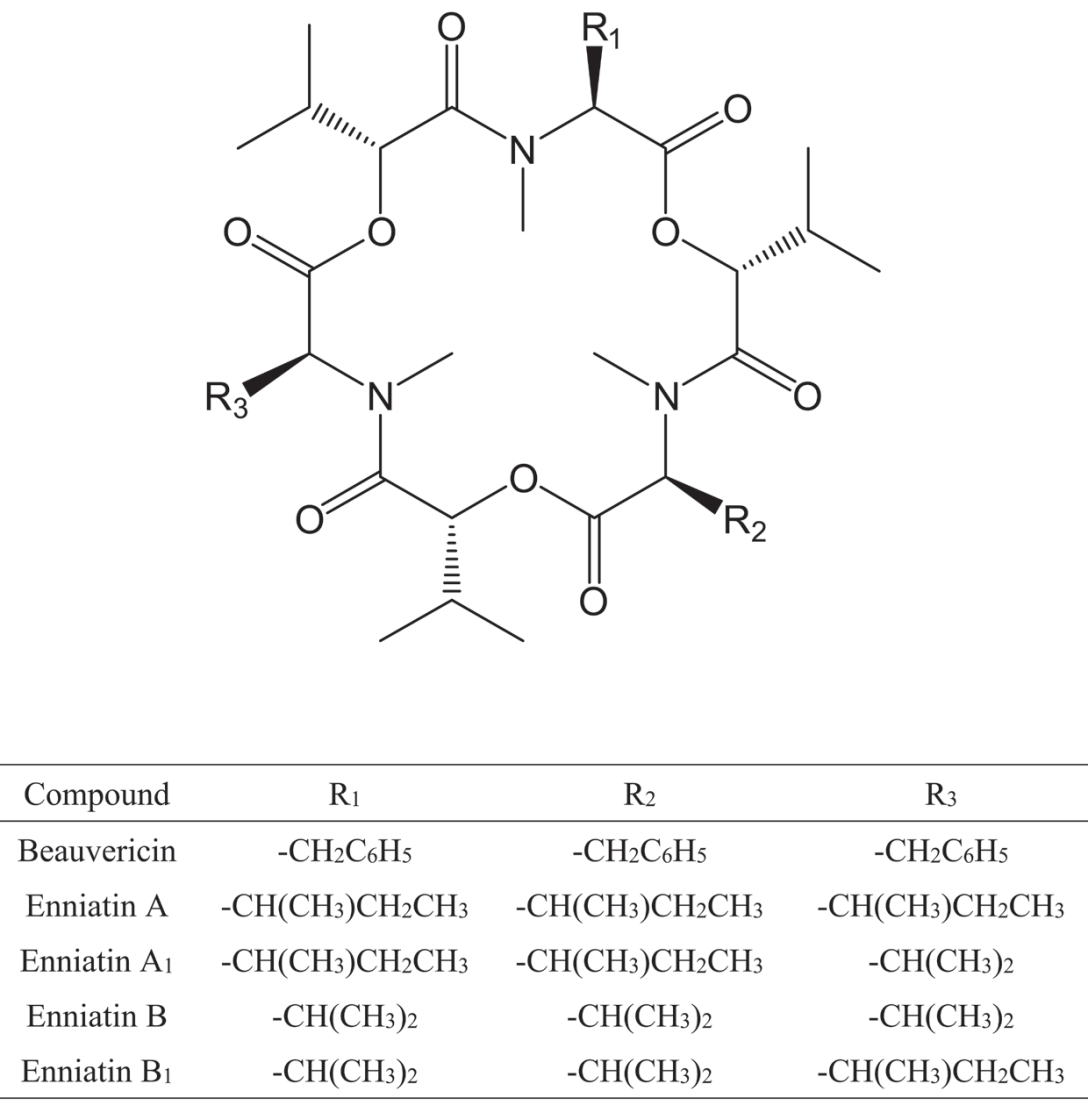
Chemical structures of beauvericin and enniatins.

BEA and ENNs are cyclic depsipeptide mycotoxins produced mainly by *Fusarium* species. BEA was first discovered in a culture of the entomopathogenic fungus, *Beauveria bassiana*^6^^)^. Subsequent studies revealed that many *Fusarium* species, such as *F. proliferatum*, *F. semitectum*, and *F. subglutinan* are producers of BEA^7^^)^. ENNs are a group of structurally related mycotoxins and approximately 30 analogs have been identified^8^^)^. They were first found in a culture of *Fusarium oxysporum*, and later, many *Fusarium* strains, such as *F. acuminatum*, *F. avenaceum* and *F. equiseti* have been shown to produce ENNs^9^^)^. Among ENNs, enniatin A, A_1_, B, and B_1_ (ENNA, ENNA_1_, ENNB, and ENNB_1_, respectively) are frequently found in food and feeds^10^^)^. The chemical structures of BEA and four ENNs are shown in Fig. 1.

Previously, we conducted a survey on the BEA and four ENNs in wheat flour and corn grits^11^^)^. In the survey, 207 samples were collected between 2014 and 2016 from the Japanese market, and the contamination levels of the five mycotoxins were determined. Among the five mycotoxins, ENNB was detected most frequently, and the positivity rates for ENNB in imported and domestic wheat flour were 81.8% and 85.6%, respectively. The highest concentration of ENNB was present in a domestic wheat flour sample (633 µg/kg). These results suggest that further studies on ENNs are required for risk assessments.

To assess the toxicity of ENNs, our group conducted a 28-day repeated oral dose toxicity study of enniatin complex (a mixture of ENNB, ENNB_1_, and ENNA_1_ at a ratio of 4:4:1) in mice^12^^)^. Slight reductions in food consumption were observed in male mice administered 4 and 20 mg/kg and in female mice administered 20 mg/kg. No other adverse events occurred at the end of the study period. Next, pharmacokinetic and 28-day repeated-dose oral toxicity studies of ENNB were performed in mice^13^^)^. The former study showed that ENNB exhibited 139.9% bioavailability, a 5.1-h elimination half-life, 5.26% fecal excretion from 4 to 24 h post-dose, and the upregulation of cytochrome P450 enzymes in the liver. In the latter study, ENNB was administered to male and female mice via oral gavage at doses of 0, 7.5, 15, and 30 mg/kg body weight/day. However, the changes observed after oral administration were minor, and the pathology and related parameters revealed no changes suggestive of ENNB. These results suggest that ENNB does not induce toxicity after 28 days of oral administration in mice, although it is well absorbed and metabolized in the liver. Another research group studied the combined effects of ENNs and deoxynivalenol (DON), a trichothecene mycotoxin, on weaning piglets^14^^)^. The diet containing both ENNB and DON significantly decreased the weight gain of the animals, whereas the diet containing ENNB or DON alone did not affect weight.

The *in vivo* toxicity of BEA and ENNs is currently unknown. However, new toxicity mechanisms, including a combinatorial effect with other mycotoxins, may be revealed in the future. To assess the health risks of ENNs, information regarding their contamination levels in food is important. In this study, an analytical method for the determination of BEA and ENNs was developed and validated in an inter-laboratory study. Furthermore, we conducted a survey of these five mycotoxins in retail foods in Japan.

## Materials and Methods

### Chemicals and samples

Solid crystals of BEA, ENNA, ENNA_1_, ENNB, and ENNB_1_ were purchased from Sigma-Aldrich (St. Louis, MO, USA). Each compound was dissolved in acetonitrile (50 mg/L each). Equal amounts of each of the five solutions were mixed to prepare a spiking solution III containing 10 mg/L of each compound. The spiking solution III was diluted with acetonitrile to prepare two spiked solutions (I, 0.5 mg/mL and II, 2 mg/L). These solutions were stored at -20 °C. LC-MS-grade acetonitrile and water, reagent-grade acetonitrile, methanol, and ammonium acetate were purchased from Wako Pure Chemical (Osaka, Japan). Food items were selected based on the results of surveys for BEA and ENNs conducted in European countries^2^^)^. Samples were purchased from local supermarkets and online shops in Japan between the summer of 2019 and winter of 2021. Granule samples including barley, Job’s tears, multigrain rice, raisin, rice, and sesame were milled into a powder using a Wander Blender WB-1 (Osaka Chemical Co., Ltd., Japan) and stored at 4 °C until analysis.

### Extraction and analysis of BEA and ENNs

Twenty grams of each food sample was weighed in a 500 mL Erlenmeyer flask with a stopper and extracted with 200 mL of acetonitrile-water (85:15, v/v) by shaking for 30 min. A portion of the extract (about 30 mL) was transferred to a 50 mL centrifuge tube and centrifuged at 1,410 × *g* for 10 min. An aliquot of the supernatant (0.4 mL) was transferred to a 1.5 mL microtube, and distilled water (0.8 mL) was added. After mixing, the microtubes were centrifuged at 12,000 × *g* for 5 min. An aliquot of the diluted extract (0.9 mL) was applied to a Sep-Pak Vac 3cc (200 mg) C18 Cartridge (Waters, Milford, MA, USA) pre-equilibrated with 3 mL of methanol and then 3 mL of water. The cartridge was washed with 3 mL of acetonitrile-water (10:90, v/v), followed by washing with 3 mL of acetonitrile-water (50:50, v/v). The toxins were eluted with 1.5 mL of acetonitrile-water (90:10, v/v). After centrifugation at 12,000 *g* for 5 min, BEA and ENNs in the eluate were quantified using a Triple Quad 4500 LC-MS/MS system (AB Sciex, Foster City, CA, USA) equipped with an ESI source and an LC-20A series HPLC system (Shimadzu Corporation, Kyoto, Japan). The column used was a 150 mm × 2.1 mm i.d., 3 μm, Inertsil ODS-3 (GL Sciences Inc., Tokyo, Japan). Chromatographic separation was achieved at 40 °C, using a gradient elution of 70−80% acetonitrile in water containing 2 mmol/L ammonium acetate from 0 to 20 min and then isocratic elution of 80% acetonitrile in water from 20 to 22 min at a flow rate of 0.2 mL/min. The injection volume was 5 μL. The ESI source was operated at 300 °C in the positive ionization mode. Other MS parameters were as follows: curtain gas at 30 psi, ion spray voltage at 5500 V, nebulizer gas (GS1) at 50 psi, turbo heater gas (GS2) at 80 psi, collision-activated dissociation gas at 9 (arbitrary units), multiple reaction monitoring, a dwell time of 250 ms, and a 5 ms pause between mass ranges. The following multiple reaction monitoring transitions were used: BEA, 801 [M + NH_4_]^+^ to 134 (quantifier ion: collision energy (CE), 95 eV) and 784 (qualifier ion: CE, 25 eV); ENNA, 699 [M + NH_4_]^+^ to 210 (quantifier ion: CE, 39 eV) and 682 (qualifier ion: CE, 25 eV); ENNA_1_, 685 [M + NH_4_]^+^ to 210 (quantifier ion: CE, 39 eV) and 668 (qualifier ion: CE, 25 eV); ENNB, 657 [M + NH_4_]^+^ to 196 (quantifier ion: CE, 37 eV) and 640 (qualifier ion: CE, 25 eV); ENNB_1_, 671 [M + NH_4_]^+^ to 196 (quantifier ion: CE, 37 eV) and 654 (qualifier ion: CE, 25 eV).

### Inter-laboratory study

Eight laboratories in Japan participated in this inter-laboratory study. Each participant in the study received the following: (a) BEA and ENNs-negative (< 1.5 µg/kg each) ground wheat for the spike-and-recovery test (180 g); (b) two pairs of naturally contaminated ground wheat (25 g each); (c) a mixed standard solution and eight spiking solutions (four pairs of spiking solutions I, II, III, and acetonitrile for blank). To evaluate recovery, 1.0 mL of each solution was added to 20.0 g of BEA- and ENN-negative ground wheat in a 500 mL flask (final concentrations, spike 1:25 μg/kg each, spike 2:100 μg/kg each, spike 3:500 μg/kg each, and blank) and kept for 30 min in the dark at room temperature. BEA and ENNs were extracted from both spiked and naturally contaminated samples and were quantified according to the above-mentioned method.

### Statistics

Data from participants were subjected to Cochran, single Grubbs, and double Grubbs tests to determine outliers^15^^)^. The relative standard deviations for repeatability (RSD_r_), reproducibility (RSD_R_), and HorRat values were obtained using analysis of variance (ANOVA) according to the AOAC guideline^16^^)^. The criterion for recovery at three spiking levels (25, 100, 500 µg/kg) was defined as 80‒110%, while those for RSD_R_ were set as <44% for spiking levels at 25 and 100 µg/kg and <32% at a spiking level at 500 µg/kg according to the procedural manual of the Codex Alimentarius Commission^17^^)^. The criteria for RSD_r_ were defined as <15% for spiking levels at 25 and 100 µg/kg and <11% at a spiking level at 500 µg/kg according to the guideline by AOAC^18^^)^.

### Performance evaluation of the analytical method for foods other than except wheat

To prepare spiked samples for the spike-recovery test, 400 µL of spiking solution I, 250 µL of spiking solution II, 1.0 mL of spiking solution II and 1.0 mL of spiking solution III were added to 20 g of ground samples to prepare the spike levels of 10, 25, 100 and 500 µg/kg, respectively. Two spiking levels were set for each food item according to the concentration of detected mycotoxins. After incubation at room temperature for 30 min, BEA and ENNs were extracted and analyzed using the method described above. Each spiked sample was analyzed in triplicate and the mean value and standard deviation were calculated.

### Survey of BEA and ENNs in food samples

The levels of mycotoxins in each food sample were analyzed using the method described above. A calibration curve was constructed to determine the concentration of each mycotoxin in the eluate obtained using the C18 cartridge. The mixed standard solution was diluted with the acetonitrile–water (90:10, v/v), and seven standard solutions of different concentrations (0.03, 0.1, 0.3, 1.0, 3.0, and 10.0 µg/L of each mycotoxin) were prepared. The concentration of each mycotoxin in the eluate was 1/50th that in the food sample. The lowest concentration of 0.03 µg/L in the calibration curve corresponds to a concentration of 1.5 µg/kg of the toxin in the food sample. The toxin concentration above 1.5 µg/kg was regarded as positive in this study. The mean concentration was calculated by setting all values below 1.5 µg/kg to zero.

## Results and Discussion

To validate the analytical method for the determination of BEA and ENNs levels in wheat, an inter-laboratory study was conducted using BEA- and ENN-negative wheat samples, three spiked samples, and two naturally contaminated wheat flour samples. The homogeneity of naturally contaminated wheat flour samples was assessed according to the International Harmonized Protocol of the International Union of Pure and Applied Chemistry (IUPAC)^19^^)^. Ten samples were randomly selected and analyzed in duplicate after each unit was split into two subfractions (10 g) (**Supplementary Table S1**). Recommendations 7 and 8, described in the IUPAC protocol, were followed for ENNs in the two naturally contaminated samples (**Supplementary Tables S2 and S3**).

Eight laboratories in Japan analyzed spiked and naturally contaminated samples in duplicate and submitted the results (Table 1‒5). None of the laboratories detected BEA or ENNs in the blank samples (data not shown). Cochran’s, single Grubbs, and paired Grubbs tests were used to identify outliers. Laboratory E and F had outliers for ENNB and ENNB_1_ in spiked samples at 100 µg/kg, but these values were not excluded so as not to exceed the maximum outlier rate of 2/9, as described in the AOAC International guidelines^16^^)^. Statistical parameters, including mean concentration, RSD_r_, RSD_R_, and HorRat values, are shown in Table 6. The mean recovery ranges of BEA, ENNA, ENNA_1_, ENNB, and ENNB_1_ were 92‒94, 94‒96, 97‒98, 98‒99, and 98‒100%, respectively. For naturally contaminated samples, only the results of ENNA, ENNB, and ENNB_1_ were evaluated because the measured values of ENNA_1_ and BEA were around the LOQ, and some laboratories did not detect them. The RSD_r_ and RSD_R_ in the spiked and naturally contaminated samples ranged from 2.1 to 5.7% and from 6.2 to 15.3%, respectively. All parameters met the criteria mentioned in the “Materials and Methods” section. HorRat values were in the range of 0.2‒0.8. According to the AOAC guidelines, the HorRat acceptability was 0.5‒2^18)^, and some results were below the lower limit. The purification procedure using a C18 cartridge was simple and all participants had sufficient experience in mycotoxin analysis. These factors were considered to have resulted in the low HorRat values.

**Table 1.** Results of the inter-laboratory study for determination of five cyclic depsipeptide mycotoxins in spiked wheat at 25 µg/kg

Laboratory ID	Concentration, µg/kg									
	Beauvericin		Enniatin A		Enniatin A_1_		Enniatin B		Enniatin B_1_	
A	20	21	22	23	23	24	23	25	25	25
B	23	23	23	24	24	24	24	24	24	25
C	22	23	20	23	22	24	24	21	23	23
D	26	26	25	26	25	26	26	26	26	26
E	20	17	21	19	21	20	22	19	21	20
F	29	29	28	27	27	28	28	29	28	28
G	26	25	25	25	26	24	25	24	25	25
H	21	21	24	22	26	25	26	25	26	25

**Table 2.** Results of the inter-laboratory study for determination of five cyclic depsipeptide mycotoxins in the spiked wheat at 100 µg/kg

Laboratory ID	Concentration, µg/kg									
	Beauvericin		Enniatin A		Enniatin A_1_		Enniatin B		Enniatin B_1_	
A	88	83	92	89	96	91	96	96	101	95
B	90	92	93	98	96	96	97	95	99	101
C	95	102	97	100	98	106	96	104	99	105
D	102	103	100	100	101	101	103	103	103	103
E	81	70	85	76	87	76	86	77	86	77
F	119	120	111	113	114	116	118	120	117	119
G	103	102	98	100	100	99	100	100	99	99
H	84	78	92	88	97	97	101	99	97	97

Underlined values indicate outliers determined by the Grubbs test.

**Table 3.** Results of the inter-laboratory study for determination of five cyclic depsipeptide mycotoxins in the spiked wheat at 500 µg/kg

Laboratory ID	Concentration, µg/kg									
	Beauvericin		Enniatin A		Enniatin A_1_		Enniatin B		Enniatin B_1_	
A	438	399	465	433	476	441	488	456	505	453
B	450	453	487	474	490	479	493	471	489	489
C	512	490	500	496	536	518	500	504	509	497
D	502	510	495	500	500	509	510	520	506	525
E	367	376	396	424	393	427	399	438	396	440
F	570	583	545	563	559	574	582	592	567	580
G	513	487	492	479	504	482	504	474	496	479
H	399	376	412	408	479	476	464	465	471	466

**Table 4.** Results of the inter-laboratory study for determination of five cyclic depsipeptide mycotoxins in the naturally contaminated wheat flour I

Laboratory ID	Concentration, µg/kg									
	Beauvericin		Enniatin A		Enniatin A_1_		Enniatin B		Enniatin B_1_	
A	0.1	0.1	0.2	0.1	4	4	135	140	37	37
B	0.2	0.2	0.1	0.1	4	4	139	132	38	37
C	0.5	0.5	0.6	0.6	5	5	146	144	38	40
D	0.2	0.2	0.2	0.2	4	4	148	143	39	37
E	< 2	< 2	< 0.3	< 0.3	4	4	129	123	35	32
F	0.3	0.3	0.5	0.4	5	5	170	165	42	40
G	< 0.5	< 0.5	< 0.5	< 0.5	4	5	146	140	38	38
H	< 1	< 1	< 1	< 1	4	4	149	149	37	38

**Table 5.** Results of the inter-laboratory study for determination of five cyclic depsipeptide mycotoxins in the naturally contaminated wheat flour II

Laboratory ID	Concentration, µg/kg									
	Beauvericin		Enniatin A		Enniatin A_1_		Enniatin B		Enniatin B_1_	
A	2	2	3	2	21	19	383	358	137	134
B	2	2	3	2	20	19	367	354	134	127
C	2	2	3	2	23	20	404	402	144	138
D	2	2	2	2	21	22	390	395	138	141
E	< 2	< 2	2	2	18	15	341	326	123	110
F	2	2	2	3	24	23	447	435	148	145
G	3	2	3	3	23	23	399	388	141	138
H	1	1	1	2	26	25	386	376	141	140

**Table 6.** Method performance data obtained from the inter-laboratory study

Sample	Analyte	Mean (µg/kg)	Recovery (%)	RSD_r_ (%)	RSD_R_ (%)	HorRat	Outliers
Spike level I (25 µg/kg)	Beauvericin	23	92	3.7	14.7	0.5	0
	Enniatin A	24	94	4.5	10.4	0.4	0
	Enniatin A_1_	24	97	3.3	9.4	0.3	0
	Enniatin B	25	98	4.1	10.1	0.4	0
	Enniatin B_1_	25	99	2.1	9.6	0.3	0
Spike level II (100 µg/kg)	Beauvericin	94	94	4.0	15.3	0.7	0
	Enniatin A	96	96	3.1	9.9	0.4	0
	Enniatin A_1_	98	98	3.7	10.0	0.4	0
	Enniatin B	99	99	3.1	10.5	0.5	2
	Enniatin B_1_	100	100	3.0	10.3	0.5	2
Spike level III (500 µg/kg)	Beauvericin	464	93	3.2	15.1	0.8	0
	Enniatin A	473	95	2.7	10.4	0.6	0
	Enniatin A_1_	490	98	3.1	9.7	0.5	0
	Enniatin B	491	98	3.3	10.1	0.6	0
	Enniatin B_1_	492	98	3.8	9.3	0.5	0
Naturally contaminated wheat flour I	Beauvericin	-	-	-	-	-	0
	Enniatin A	-	-	-	-	-	0
	Enniatin A_1_	4	-	2.9	10.8	0.3	0
	Enniatin B	144	-	2.4	8.7	0.4	0
	Enniatin B_1_	38	-	2.9	6.2	0.2	0
Naturally contaminated wheat flour II	Beauvericin	-	-	-	-	-	0
	Enniatin A	2	-	16.5	22.7	0.6	0
	Enniatin A_1_	21	-	5.7	12.9	0.5	0
	Enniatin B	384	-	2.4	8.4	0.5	0
	Enniatin B_1_	136	-	3.0	7.1	0.3	0

RSD_r_: repeatability relative standard deviation, RSD_R_: reproducibility relative standard deviation

The suitability of this method for the analysis of BEA and ENNs in other foods was confirmed by spike-recovery tests. The method was applied to the analysis of ten spiked food items containing five analytes in triplicate. The recoveries of the five analytes from the spiked samples are listed in Table 7. The mean recovery ranges for BEA, ENNA, ENNA_1_, ENNB, and ENNB_1_ were 87‒111, 84‒107, 83‒106, 91‒109, and 90‒106%, respectively. Except for the result of the spiked soybean flour sample at 100 µg/kg, the mean recoveries were in the range of 80-110%. These results indicate that the analytical method was successfully validated by an interlaboratory study and was suitable for determining BEA and ENNs in 11 foods. A spike-recovery test was not performed for multigrain rice. Multigrain rice is a mixture of rice, barley, Job’s tears, corn and other cereals, and spike recovery tests was conducted for each cereal.

**Table 7.** Recovery of five cyclic depsipeptide mycotoxins from each spiked commodity

Commodity	Spiking level (µg/kg)	Mean recovery ± relative standard deviation (%)														
		Beauvericin			Enniatin A			Enniatin A_1_			Enniatin B			Enniatin B_1_		
Barley	25	106	±	4.1	103	±	6.8	102	±	3.9	100	±	5.7	101	±	6.3
	100	109	±	3.5	105	±	2.7	104	±	2.0	104	±	3.2	103	±	1.8
Buckwheat flour	25	89	±	1.6	88	±	1.8	83	±	1.7	96	±	3.7	93	±	3.5
	100	87	±	0.9	89	±	0.1	84	±	0.6	91	±	0.3	90	±	0.4
Coffee	25	98	±	0.6	97	±	1.0	96	±	1.5	97	±	2.1	97	±	1.1
	100	99	±	4.4	95	±	2.2	96	±	1.7	97	±	1.9	97	±	1.3
Corn flour	25	99	±	5.7	98	±	3.3	98	±	3.2	99	±	2.8	100	±	2.6
	100	94	±	3.5	93	±	3.4	96	±	1.9	96	±	2.1	96	±	2.1
Job’s tears	25	100	±	0.7	95	±	1.7	100	±	2.7	109	±	8.6	103	±	3.7
	100	90	±	1.9	84	±	0.7	91	±	1.3	96	±	1.6	95	±	1.6
Raisin	10	104	±	2.1	102	±	0.8	106	±	2.9	105	±	6.7	102	±	4.3
	100	97	±	0.4	100	±	0.3	98	±	1.7	99	±	0.2	99	±	2.0
Rice	25	107	±	1.8	102	±	0.9	102	±	3.4	103	±	2.6	101	±	0.9
	100	106	±	0.9	101	±	1.6	101	±	1.1	101	±	0.9	99	±	0.8
Rye flour	100	102	±	1.1	100	±	2.3	102	±	3.0	103	±	1.2	104	±	3.4
	500	102	±	1.9	99	±	1.8	99	±	2.0	100	±	1.9	102	±	1.3
Sesame	10	98	±	2.5	95	±	0.6	99	±	2.9	105	±	0.4	103	±	2.6
	100	97	±	1.2	100	±	2.2	97	±	2.2	102	±	3.0	98	±	1.5
Soybean flour	10	107	±	5.1	100	±	4.2	104	±	6.0	109	±	4.9	105	±	6.9
	100	111	±	4.7	107	±	4.1	106	±	3.2	108	±	3.5	106	±	4.1

A total of 658 food samples were collected over three years, and the contamination levels of BEA and ENNs were examined using the validated analytical method. Table 8 shows the results of the survey, including the number of samples, the number and percentage of positive samples, and the mean and maximum concentrations of BEA and ENNs. BEA, ENNA, ENNA_1_, ENNB, and ENNB_1_ were detected in 153 (23%), 43 (7%), 115 (17%), 264 (40%), and 215 (33%) samples, respectively. BEA was detected in corn flour (76%), Job’s tears (64%), and soybean flour (50%). The mean BEA value was highest in soybean flour (13 µg/kg), followed by Job’s tears (10 µg/kg) and corn flour (6 µg/kg). The maximum concentration level of BEA was detected in a Job’s tears sample (128 µg/kg). In a report published by the EFSA, the occurrence of BEA in cereals from European countries was summarized^2^^)^. For example, in wheat samples collected in the Mediterranean area in 2010, BEA was detected in 3 out of 21 samples (14%) at the concentration range of 2.4‒61.4 µg/kg^20^^)^. In our wheat flour samples, the positive rate, the mean value and the maximum concentration of BEA were 17%, 0.7 µg/kg and 11 µg/kg, respectively. The contamination level of BEA in the Mediterranean wheat flour samples was similar to our samples. In maize samples collected from Germany in 2007, the mean and maximum concentrations of BEA were 240 µg/kg and 5,100 µg/kg, respectively^21^^)^. The contamination level of BEA in German maize samples was higher than that in our corn flour samples.

**Table 8.** Occurrence of five cyclic depsipeptide mycotoxins in foods retailed in Japan

Commodity	No. of sample	Beauvericin				Enniatin A				Enniatin A_1_				Enniatin B				Enniatin B_1_		
		No. of positive samples (%)	Mean (µg/kg)	Maximum (µg/kg)		No. of positive samples (%)	Mean (µg/kg)	Maximum (µg/kg)		No. of positive samples (%)	Mean (µg/kg)	Maximum (µg/kg)		No. of positive samples (%)	Mean (µg/kg)	Maximum (µg/kg)		No. of positive samples (%)	Mean (µg/kg)	Maximum (µg/kg)
Barley	20	0 (0)	-	-		0 (0)	-	-		1 (5)	0	2		6 (30)	3	30		5 (25)	1	8
Buckwheat flour	37	2 (5)	0.2	5		0 (0)	-	-		0 (0)	-	-		0 (0)	-	-		0 (0)	-	-
Coffee	60	1 (2)	0.1	7		0 (0)	-	-		0 (0)	-	-		3 (5)	0	3		0 (0)	-	-
Corn flour	21	16 (76)	6	20		0 (0)	-	-		0 (0)	-	-		0 (0)	-	-		0 (0)	-	-
Job’s tears	88	56 (64)	10	128		0 (0)	-	-		0 (0)	-	-		1 (1.1)	0	8		1 (1.1)	0	2
Multigrain rice	20	7 (35)	5	43		0 (0)	-	-		0 (0)	-	-		4 (20)	3	43		3 (15)	1	8
Raisin	11	0 (0)	-	-		0 (0)	-	-		0 (0)	-	-		0 (0)	-	-		0 (0)	-	-
Rice	60	2 (3)	0.2	9		0 (0)	-	-		0 (0)	-	-		3 (5)	0	4		1 (2)	0	2
Rye flour	121	15 (12)	1	50		27 (22)	3	115		68 (56)	32	1,468		105 (87)	987	48,800		94 (78)	256	12,589
Sesame	30	12 (40)	3	19		0 (0)	-	-		0 (0)	-	-		0 (0)	-	-		0 (0)	-	-
Soybean flour	30	15 (50)	13	101		1 (3)	0	3		1 (3)	0	5		18 (60)	3	24		8 (27)	1	14
Wheat flour	160	27 (17)	0.7	11		15 (9)	0	14		45 (28)	2	30		124 (78)	49	830		103 (64)	15	233
Overall	658	153 (23)				43 (7)				115 (17)				264 (40)				215 (33)		

Among the four ENNs, ENNB contamination level was the highest among all food samples. ENNB was mainly detected in rye flour (87%), wheat flour (78%), and soybean flour (60%). The mean ENNB value was highest in rye flour (987 µg/kg), followed by wheat flour (49 µg/kg). A sample with an extreme contamination level was found in rye flour (48,800 µg/kg). The maximum concentration in wheat samples was 830 µg/kg. The ENNB contamination levels in foods other than rye flour and wheat flour were relatively low. In a survey conducted in European countries, high levels of ENNs contamination were observed in wheat and barley. Similar to our samples, among the four ENNs, the ENNB contamination level was the highest in the European samples. For example, the mean and maximum ENNB concentrations in spring wheat samples collected in Sweden in 2011 were 169 and 984 µg/kg, respectively^22^^)^. In barley samples collected in Finland between 2001 and 2002, the range of detected ENNB was 44‒9,760 µg/kg^23^^)^.

In this study, an analytical method for the determination of BEA and four ENNs was developed and their occurrence in 12 foods was investigated. ENNB was the most frequently detected among the five mycotoxins and was mainly found in rye flour and wheat flour. Wheat flour is a potential source of ENNs exposure in Japan because it is a staple food. This information is important for evaluating the risks of ENNs to human health. However, it is difficult to determine the risk at this time, because their toxicity in mammals remains unknown. Further studies on their toxicity, including combination toxicity with other mycotoxins, are needed to perform more precise risk assessment for BEA and ENNs.

## Supplementary materials
