# Supplementary material for: Study About Beauvericin and Enniatins: Method Validation and Survey for Foods in Japan
Source: Food Saf (Tokyo). 2025 Dec 19;13(4):91–100. doi: 10.14252/foodsafetyfscj.D-25-00018 (PMC12718107; doi:10.14252/foodsafetyfscj.D-25-00018)
Supplement: Supplementary file 1 [file foodsafetyfscj-13-4-91-s001.pdf]

## Supplementary materials

**Supplementary Table S1.** Analysis of five cyclic depsipeptide mycotoxins in naturally contaminated wheat flour I for verification of homogeneity

| No. | Concentration (μg/kg) | | | | | | | | | |
|---|---|---|---|---|---|---|---|---|---|---|
| | Beauvericin | | Enniatin A | | Enniatin $A_1$ | | Enniatin B | | Enniatin $B_1$ | |
| 1 | < 1.5 | < 1.5 | < 1.5 | < 1.5 | 4.2 | 4.5 | 146 | 145 | 39 | 40 |
| 2 | < 1.5 | < 1.5 | < 1.5 | < 1.5 | 4.6 | 4.6 | 152 | 150 | 41 | 40 |
| 3 | < 1.5 | < 1.5 | < 1.5 | < 1.5 | 4.5 | 4.9 | 148 | 151 | 40 | 41 |
| 4 | < 1.5 | < 1.5 | < 1.5 | < 1.5 | 4.5 | 5.0 | 151 | 148 | 42 | 39 |
| 5 | < 1.5 | < 1.5 | < 1.5 | < 1.5 | 4.8 | 5.0 | 154 | 153 | 41 | 41 |
| 6 | < 1.5 | < 1.5 | < 1.5 | < 1.5 | 4.8 | 4.6 | 147 | 153 | 41 | 41 |
| 7 | < 1.5 | < 1.5 | < 1.5 | < 1.5 | 4.7 | 4.7 | 150 | 155 | 40 | 42 |
| 8 | < 1.5 | < 1.5 | < 1.5 | < 1.5 | 4.7 | 5.2 | 156 | 156 | 41 | 42 |
| 9 | < 1.5 | < 1.5 | < 1.5 | < 1.5 | 4.7 | 4.9 | 153 | 155 | 40 | 42 |
| 10 | < 1.5 | < 1.5 | < 1.5 | < 1.5 | 5.3 | 4.9 | 157 | 158 | 43 | 42 |

**Supplementary Table S2.** Analysis of five cyclic depsipeptide mycotoxins in naturally contaminated wheat flour II for verification of homogeneity

| No. | Concentration (μg/kg) | | | | | | | | | |
|---|---|---|---|---|---|---|---|---|---|---|
| | Beauvericin | | Enniatin A | | Enniatin $A_1$ | | Enniatin B | | Enniatin $B_1$ | |
| 1 | < 1.5 | < 1.5 | 2.2 | 2.8 | 23 | 24 | 407 | 394 | 140 | 136 |
| 2 | < 1.5 | < 1.5 | 2.3 | 2.8 | 22 | 23 | 416 | 408 | 142 | 141 |
| 3 | < 1.5 | < 1.5 | 2.3 | 3.0 | 23 | 24 | 418 | 404 | 142 | 147 |
| 4 | < 1.5 | < 1.5 | 2.5 | 3.0 | 23 | 23 | 421 | 402 | 148 | 142 |
| 5 | < 1.5 | < 1.5 | 3.0 | 3.1 | 24 | 25 | 431 | 436 | 148 | 154 |
| 6 | < 1.5 | < 1.5 | 3.2 | 3.1 | 25 | 26 | 438 | 444 | 152 | 153 |
| 7 | < 1.5 | < 1.5 | 3.2 | 2.9 | 24 | 25 | 419 | 423 | 152 | 154 |
| 8 | < 1.5 | < 1.5 | 2.9 | 3.2 | 25 | 24 | 419 | 415 | 152 | 149 |
| 9 | < 1.5 | < 1.5 | 3.0 | 2.8 | 25 | 25 | 397 | 406 | 145 | 149 |
| 10 | < 1.5 | < 1.5 | 2.8 | 3.3 | 25 | 25 | 399 | 401 | 146 | 148 |

**Supplementary Table S3.** Assessment for the homogeneity of naturally contaminated wheat according to Recommendation 7* in the International Harmonized Protocol of IUPAC

| Naturally contaminated wheat flour | Analyte | $\sigma_{an}$ | $\sigma_p$ | $\sigma_{an} / \sigma_p$ |
|---|---|---|---|---|
| I | Enniatin $A_1$ | 0.227 | 1.05 | 0.22 |
| | Enniatin B | 1.971 | 32.3 | 0.06 |
| | Enniatin $B_1$ | 1.05 | 9.00 | 0.12 |
| II | Enniatin $A_1$ | 0.287 | 0.633 | 0.45 |
| | Enniatin $A_1$ | 0.525 | 5.31 | 0.10 |
| | Enniatin B | 6.92 | 75.8 | 0.09 |
| | Enniatin $B_1$ | 2.69 | 31.4 | 0.09 |

* Recommendation 7
The analytical (repeatability) precision of the method used in the homogeneity test ($\sigma_p$) should satisfy $\sigma_{an}/\sigma_p < 0.5$ where $\sigma_{an}$ is the repeatability standard deviation appropriate to the homogeneity test.

**Supplementary Table S4.** Assessment for the homogeneity of naturally contaminated wheat according to Recommendation 8* in the International Harmonized Protocol of IUPAC

| Naturally contaminated wheat flour | Analyte | $S_{sam}$^2 | F1 ×$\sigma_{all}$^2 + F2 × $S_{an}$^2 |
|---|---|---|---|
| I | Enniatin $A_1$ | 0.0167 | 0.237 |
| | Enniatin B | 11.1 | 180 |
| | Enniatin $B_1$ | 0.000 | 14.8 |
| II | Enniatin $A_1$ | 0.0118 | 0.151 |
| | Enniatin $A_1$ | 0.905 | 5.06 |
| | Enniatin B | 167 | 1019 |
| | Enniatin $B_1$ | 20.0 | 174 |

* Recommendation 8
Employ an explicit test of the hypothesis H: $\sigma_{sam}$^2 ≤ $\sigma_{all}$^2, by finding a one-sided 95% confidence interval for $\sigma_{sam}$^2 and rejecting H when this interval does not include $\sigma_{sam}$^2.
This is equivalent to rejecting H when $S_{sam}$^2 > F1×$\sigma_{all}$^2 + F2×$S_{an}$^2
where $S_{sam}$^2 and $S_{an}$^2 are the usual estimates of sampling and analytical variances obtained from the ANOVA, and F1 and F2 are constants that may be derived from standard statistical tables.
In our study, the values of F1 and F2 were 1.88 and 1.01, respectively, because 10 test samples were analyzed in duplicate.
